# Supplementary material for: Comparing Executive Functions in Children and Adolescents with Autism and ADHD—A Systematic Review and Meta-Analysis
Source: Children (Basel). 2024 Apr 15;11(4):473. doi: 10.3390/children11040473 (PMC11049008; doi:10.3390/children11040473)
Supplement: Supplementary file 1 [file children-11-00473-s001.zip › children-2931659-supplementary.pdf]

Table S1. Summary of details of the included Studies - Neuropsychological Test

Study (1st author, year)	Sample Size (Male/Female)	Mean Age M (SD)	Mean IQ M (SD)	Double Diagnosis Yes/No	Med.	EFs components	EFs Test
Corbett, 2009	ADHD 12/6<br>ASD17/6<br>TD12/6	ADHD 9.4(1.98)<br>ASD 9.44(1.96)<br>TD 9.56(1.81)	ADHD 105.17(12.82)<br>ASD 94.17(17.79)<br>TD 112.22(14.84)	No	No W/O	v-s WM<br>Inhib.<br>Flex<br>Plan	SWM-CANTAB<br>Stroop<br>D-K T-Switch, IED, CCTT<br>SoC
Boxhorn, 2018	ADHD 58/0<br>ASD 23/0<br>TD 22/0	ADHD 9.76(1.8)<br>ASD 10.04(2.3)<br>TD 9.77 (2.4)	ADHD 102.33(11.2)<br>ASD 104.96(18.3)<br>TD 112(13)	No	W/O	Inhib	GNG
Gaizhi-Li, 2017	ADHD 58/0<br>ASD 32/0<br>TD 39/0	ADHD 10.38(2.29)<br>ASD 10.31(3.34)<br>TD 10.72(2.21)	ADHD 99.26(11.79)<br>ASD 94.44(20.53)<br>TD 111.38(11.29)	No	W/O	v-WM<br>Prob-Solv	B-Digit Span<br>Raven Matrices,<br>Block Design
Geurts, 2004	ADHD 54/0<br>ASD 41/0<br>TD 41/0	ADHD 9.3(2)<br>ASD 9.4(1.8)<br>TD 9.1(1.7)	ADHD 99.5(11.5)<br>ASD 98.3(18.4)<br>TD 115.5(18)	No	W/O	Inhib.<br>v-s WM<br>Plan<br>Flex	GNG,Circle Drawing, OW<br>SoP<br>ToL<br>WCST
Goldberg, 2005	ADHD 19/2<br>ASD 13/4<br>TD 21/11	ADHD 9.8(1.3)<br>ASD 10.3(1.8)<br>TD 10.4(1.5)	ADHD 113.8(10.3)<br>ASD 96.5(15.9)<br>TD 112.6(12.1)	No	W/O	Inhib<br>Plan<br>Flex<br>v-s WM	Stroop<br>SoC<br>IED<br>SWM-CANTAB
Gomarus,2008	ADHD 11/4<br>ASD 13/2<br>TD 11/4<br>ADHD+ASD 10/5	ADHD 9.82(1.09)<br>ASD 10.25(1.11)<br>TD 10.15(1.41)<br>ADHD+ASD 10.13(1.33)	ADHD 103.5(10.2)<br>ASD 108.6(17.5)<br>TD 107.1(15.6)<br>ADHD+ASD 101.9(14)	Yes	W/O	v-s WM	Visual Search
Hutchison,2016	ADHD 13/8<br>ASD 32/1<br>TD 19/9	ADHD 11(2.3)<br>ASD 11.64(2.8)<br>TD 10.43(3.22)	ADHD 107.1(14.8)<br>ASD 112.2(14.4)	No	W/O	Flex	WCST
Hwang-Gu, 2019	ADHD 82/16<br>ASD 110/14<br>TD 208/41<br>ADHD+ASD 80/17	ADHD 10.94(2.34)<br>ASD 11.49(2.58)<br>TD 11.53(2.56)<br>ADHD+ASD 11.83(2.6)	ADHD 102.87(13.18)<br>ASD 108.78(14.15)<br>TD 113.11(10.47)<br>ADHD+ASD 104.01(13.54)	Yes		Inhib	CPT
Johnson,2007	ADHD 20/3<br>ASD 20/1<br>TD 15/3	ADHD 10.5(2.4)<br>ASD 12.2(2.4)<br>TD 11.1(1.9)	ADHD 98.7(14.6)<br>ASD 97.3(12.3)<br>TD 107.7(11.6)	No	No W/O	Inhib	GNG

Table S1. Continued

Study (1st author, year)	Sample Size (Male/Female)	Mean Age M (SD)	Mean IQ M (SD)	Double Diagnosis Yes/No	Med.	EFs components	EFs Test
Mahone,2006	ADHD 40/12<br>ASD 23/1<br>TD 34/26	ADHD 9.3(1.2)<br>ASD 10(1.6)<br>TD 9.8(1.4)	ADHD 109.7(12.6)<br>ASD 99.1(16.3)<br>TD 118.2(10.5)	No	No W/O	Inhib	CMRT, NEPSY-statue
Matsuura,2014	ADHD 13/2<br>ASD 11/0<br>TD 12/7	ADHD 10.8(1.8)<br>ASD 12(2.2)<br>TD 11.4(1.6)	ADHD 103.8(14.9)<br>ASD 105.6(14.3)<br>TD 111.8(13.4)	No	W/O	v-s WM<br>Prob-Solv	SWM-CANTAB<br>Block Design, Raven Matrices
Nydén,1999	ADHD 10/0<br>ASD 10/0<br>TD 10/0	ADHD 10(0.9)<br>ASD 10.1(0.9)<br>TD 10(0.9)	ADHD 91.2(13.11)<br>ASD 100.7(16.3)<br>TD	No		Inhib<br>Flex	GNG<br>WCST
Ozonoff,1999	ADHD 40/<br>ASD 24/<br>TD 29/	ADHD 11.1(2)<br>ASD 12.6(3.4)<br>TD 12.1(3)	ADHD 107.2(10.8)<br>ASD 95.2(18.8)<br>TD 107.8(10.8)	No		Flex<br>Plan<br>Inhib	WCST<br>Toh<br>Stroop
Karalunas,2018	ADHD 359/150<br>ASD 84/13<br>TD 146/155	ADHD 9.72(1.59)<br>ASD 11.34(2.49)<br>TD 9.48 (1.59)	ADHD 108.27(13.88)<br>ASD 99.53(16.37)<br>TD 115.28(12.75)	No	W/O	v-s WM<br>Flex<br>Inhib	SSP-CANTAB<br>TMT<br>Stroop, GNG
Pitzianti,2016	ADHD 12/1<br>ASD 13/0<br>TD 12/1<br>ADHD+ASD 10/2	ADHD10.15(1.9)<br>ASD 10.69(2.1)<br>TD 11.85(2.7)<br>ADHD+ASD 10.25(2)	ADHD 97.46(11.4)<br>ASD 106(17.41)<br>TD 106.31(11.84)<br>ADHD+ASD 108.36(14.84)	Yes	W/O	Plan<br>v WM<br>Inhib	ToL<br>B-Digit Span<br>GNG
Salunkhe,2018	ADHD 35/5<br>ASD 19/2<br>TD 35/5<br>ADHD+ASD 16/3	ADHD 10.29(1.44)<br>ASD 10.79(1.66)<br>TD 10.23(1.58)<br>ADHD+ASD 9.98(1.77)	ADHD 104.73(18.74)<br>ASD 109.1(21.12)<br>TD 107.2(18.33)<br>ADHD+ASD 103.95(19.43)	Yes	W/O	v-s WM<br>Inhib	NBT<br>GNG
Samyn,2015	ADHD 30/0<br>ASD 31/0<br>TD 95/53	ADHD 13.16(1.61)<br>ASD 12.83(1.41)<br>TD 12.73(1.48)	ADHD 108.2(12.63)<br>ASD 101.16(12.48)<br>TD 107.21 (11.68)	No	W/O	Inhib	GNG, Stroop
Samyn,2013	ADHD 25/0<br>ASD 25/0<br>TD 25/0	ADHD 13.16(1.61)<br>ASD 12.75(1.49)<br>TD 12.94(1.45)	ADHD 107.92(12.11)<br>ASD 101.24(11.45)<br>TD 104.92(10.23)	No	W/O	Inhib	Flanker
Semrud-Clikeman,2010	ADHD 14/7<br>ASD 8/7<br>TD 23/9	ADHD 10(1.9)<br>ASD 10.6(2.6)<br>TD 9.8(2.1)	ADHD 109.8(13.3)<br>ASD 100.8(13)<br>TD 109.4(10)	No	W/O	Plan<br>Inhib<br>Prob-Solv	Tower Task D-KEFS<br>Stroop<br>Fluid Reasoning-WJ COG III

Table S1. Continued

Study<br>(1st author, year)	Sample Size<br>(Male/Female)	Mean Age<br>M (SD)	Mean IQ<br>M (SD)	Double Diagnosis<br>Yes/No	Med.	EFs components	EFs Test
Semrud-Clikeman,2010b	ADHD 76/<br>ASD 50/<br>TD 113/	ADHD 10(24.4)<br>ASD 10.3(28.8)<br>TD 10.4(25.1)	ADHD 110(16.6)<br>ASD 107.3(17.6)<br>TD 111.9(14.3)	No	W/O	Prob-Solv	Fluid Reasoning-WJ COG III
Sinzig, 2008	ADHD 19/1<br>ASD 16/4<br>TD 14/6<br>ADHD+ASD 19/1	ADHD 12.2(2)<br>ASD 14.3(3)<br>TD 13.1(3)<br>ADHD+ASD 10.9(3.1)	ADHD 98(13.4)<br>ASD 112(17.7)<br>TD 113(11.9)<br>ADHD+ASD 103(13)	Yes	W/O	Inhib<br>Flex<br>v-s WM<br>Plan	GNG<br>IED<br>SWM-CANTAB<br>SoC
Sinzig,2008b	ADHD27/3<br>ASD 16/4<br>TD 23/7<br>ADHD+ASD 20/1	ADHD 12.9(3.1)<br>ASD 14.5(3)<br>TD 12.8(2.8)<br>ADHD+ASD 10.7(3.2)	ADHD 102(15.8)<br>ASD 112(19)<br>TD 109(12.8)<br>ADHD+ASD 103(13)	Yes	W/O	Inhib	GNG
Tsuchiya,2004	ADHD 20/2<br>ASD 16/1<br>TD 13/12	ADHD 11.3(2.6)<br>ASD 12.5(4.3)<br>TD 12.7(3.1)	ADHD 98.3(15.1)<br>ASD 92.3(12.5)<br>TD	No		Flex	WCST
Tye,2013	ADHD 18/0<br>ASD 19/0<br>TD 26/0<br>ADHD+ASD 29/0	ADHD 10.48(1.91)<br>ASD 11.69(1.7)<br>TD 10.56(1.79)<br>ADHD+ASD 10.53(1.69)	ADHD 104.11(14.23)<br>ASD 115.68(15.73)<br>TD 120.04(13.42)<br>ADHD+ASD 109.72(13.41)	Yes	W/O	Inhib	CPT
Unterrainer,2016	ADHD 42/0<br>ASD 18/0<br>TD 42/0<br>ADHD+ASD 23/0	ADHD 9.83(2.15)<br>ASD 10.14(2.44)<br>TD 9.76(2.36)<br>ADHD+ASD 10.17(2.05)	ADHD 94.46(14.29)<br>ASD 97.08(16.42)<br>TD 97.59(13.86)<br>ADHD+ASD 98.85(12.51)	Yes	W/O	Plan	ToL
Verté,2006	ADHD 54/11<br>ASD 61/5<br>TD 67/15	ADHD 9.1(2)<br>ASD 8.7(2)<br>TD 9.2(1.7)	ADHD 99.8(11.7)<br>ASD 101.5(18.2)<br>TD 112.2(16)	No	W/O	Inhib<br>v-s WM	GNG<br>SoP
Wang,2018	ADHD 23/5<br>ASD 20/1<br>TD 19/9	ADHD 8.18(1.93)<br>ASD 9.05(2.38)<br>TD 8.92(1.68)	ADHD 93.25(7.23)<br>ASD 99.53(17.78)<br>TD 110.14(11.91)	No	W/O	Flex<br>v-s WM	WCST<br>CBTT
Xiao,2012	ADHD 16/0<br>ASD 19/0<br>TD 16/0	ADHD 9.75(1.8)<br>ASD 10.11(2.08)<br>TD 9.69(1.74)	ADHD 103.63(8.13)<br>ASD 99.26(9.03)<br>TD 105.63(13.12)	No		Inhib	GNG, Stroop

Table S1. Continued

Study (1st author, year)	Sample Size (Male/Female)	Mean Age M (SD)	Mean IQ M (SD)	Double Diagnosis Yes/No	Med.	EFs components	EFs Test
Yasumura,2014	ADHD 8/2<br>ASD 7/4<br>TD 6/9	ADHD 11.8(2.23)<br>ASD 10.51(2.3)<br>TD 9.56(1.51)	ADHD > 80<br>ASD > 80<br>TD > 80	No		Inhib	Stroop
Azadi Sohi,2012	ADHD 35/0<br>ASD 19/0<br>TD 42/0<br>ADHD+ASD 24/0	ADHD 11.02(2.66)<br>ASD 11.12(1.64)<br>TD 10.55(2.43)<br>ADHD+ASD 10.63(2.13)	ADHD 100.69(14.16)<br>ASD 111.47(15.98)<br>TD 120.83(13.06)<br>ADHD+ASD 105.64(13.14)	Yes	W/O	Inhib	GNG
Manteris,2013	ADHD 12/8<br>ASD 15/3<br>TD 8/13	ADHD 9.06(1.18)<br>ASD 8.84(1.59)<br>TD 8.96(1.31)	ADHD 116.55(13.21)<br>ASD 102.33(14.87)<br>TD 116.33(12.87)	No	W/O	Flex<br>Inhib	WCST<br>GNG
Shahabbudin,2015	ADHD 9/7<br>ASD 12/4<br>TD 9/7<br>ADHD+ASD 15/1	ADHD 8.89(0.72)<br>ASD 9.68(0.81)<br>TD 9.37(0.84)<br>ADHD+ASD 9.71(0.86)	ADHD 103.55(10.26)<br>ASD 101.75(12.11)<br>TD 101.18(2.97)<br>ADHD+ASD 99.47(8.82)	Yes		Inhib	CPT

Note. Medication: W/O= no medication/ adequate period of washout from all medications; No W/O= inadequate period of washout/partial washout;

EFs components: v-sWM= visuo spatial WM; vWM= verbal WM; inhib= inhibition; Flex= cognitive flexibility; Plan=planning; Prob-Solv= problem-solving;

Tasks: SWM= spatial working memory (CANTAB); D-K T switch= Delis-Kaplan Executive Function System- total switching; B-Digit Span = backward digit span (Wechsler Intelligence Scale for Children, IV); IED= Intradimensional-Extradimensional Shift of CANTAB; CCTT= Child Color Trail Test; SoC= Stocking of Cambridge; GNG= Go/No-Go; OW= opposite worlds of TEA-ch; SoP= self-ordered point; ToL= tower of London; WCST= Wisconsin Card Sorting Test; CMRT= conflicting motor response task and contralateral motor response task; TMT=trail making test; ToH=tower of Hanoi; NBT= N-Back Task; WJ COG III= Fluid intelligence reasoning of the Woodcock-Johnson Cognitive Battery III; CBTT= Corsi Block Tapping Test.

Table S2 | Summary of details of the included Studies - Questionnaires

Study (1st author, year)	Sample Size (Male/Female)	Mean Age M (SD)	Mean IQ M (SD)	Double Diagnosis Yes/No	Med.	EFs Questionnaire	Rater (Parent/Teacher/Children)
Berenguer,2018	ADHD 33/2<br>ASD 27/2<br>TD 23/14<br>ADHD+ASD 20/2	ADHD 9.14(1.4)<br>ASD 8.39(1.3)<br>TD 8.54(1.2)<br>ADHD+ASD 8.86(1.3)	ADHD 99.03(9.8)<br>ASD 100.37(12.4)<br>TD 102.11(8.9)<br>ADHD+ASD 102.86(13)	Yes	No W/O	BRIEF	Teacher
Hovik,2017	ADHD 20/13<br>ASD 28/6<br>TD 32/18	ADHD 11.6(2.1)<br>ASD 11.9(2.3)<br>TD 11.6(2)	ADHD 96.8(13.8)<br>ASD 98.2(18.6)<br>TD 103.8(12.9)	No	W/O	BRIEF	Parent
Samyn,2011	ADHD 27/0<br>ASD 27/0<br>TD 27/0	ADHD 13.21(1.57)<br>ASD 12.73(1.46)<br>TD 12.91(1.43)	ADHD 107.81(13.21)<br>ASD 102.22(11.69)<br>TD 105.04(10.71)	No	W/O	EATQ-R; ACS	Parent; Children
Samyn,2015	ADHD 30/0<br>ASD 31/0<br>TD 95/53	ADHD 13.16(1.61)<br>ASD 12.83(1.41)<br>TD 12.73(1.48)	ADHD 108.2(12.63)<br>ASD 101.16(12.48)<br>TD 107.21(11.68)	No	W/O	EATQ-R; ACS	Parent, Children
Semrud-Clikeman, 2010	ADHD 14/7<br>ASD 8/7<br>TD 23/9	ADHD 10(1.9)<br>ASD 10.6(2.6)<br>TD 9.8(2.1)	ADHD 109.8(13.3)<br>ASD 100.8(13)<br>TD 109.4(10)	No	W/O	BRIEF	Parent

Note. BRIEF= Behavior Rating Inventory of Executive Function; EATQ-R= Early Adolescent Temperament Questionnaire-Revised; ACS= Attentional Control Scale

Publication Bias

Figure S1. Funnel plot ADHD vs ADHD+ASD

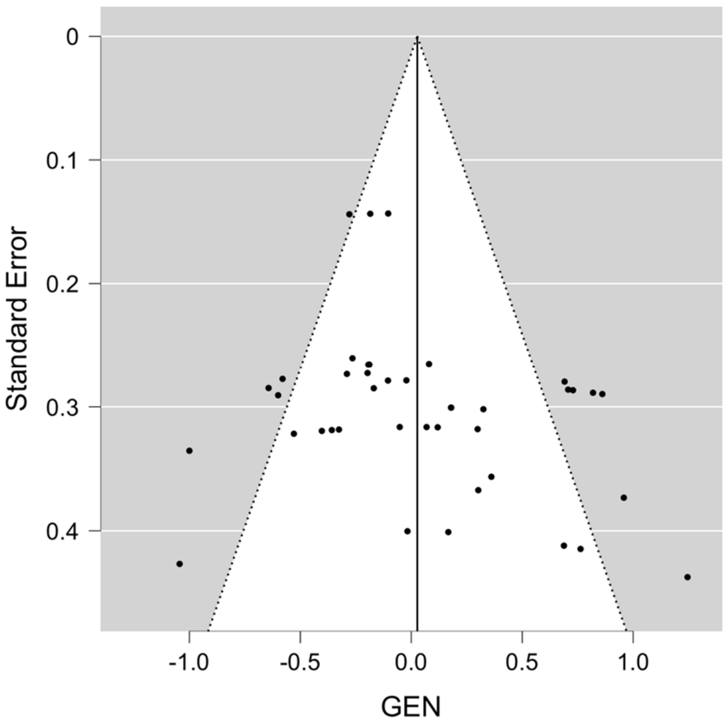

Rank Correlation test for funnel plot asymmetry shows no indication of publication bias (Kendall's tau=0.183, *p*=0.094)

Figure S2. Funnel plot ASD vs ADHD+ASD

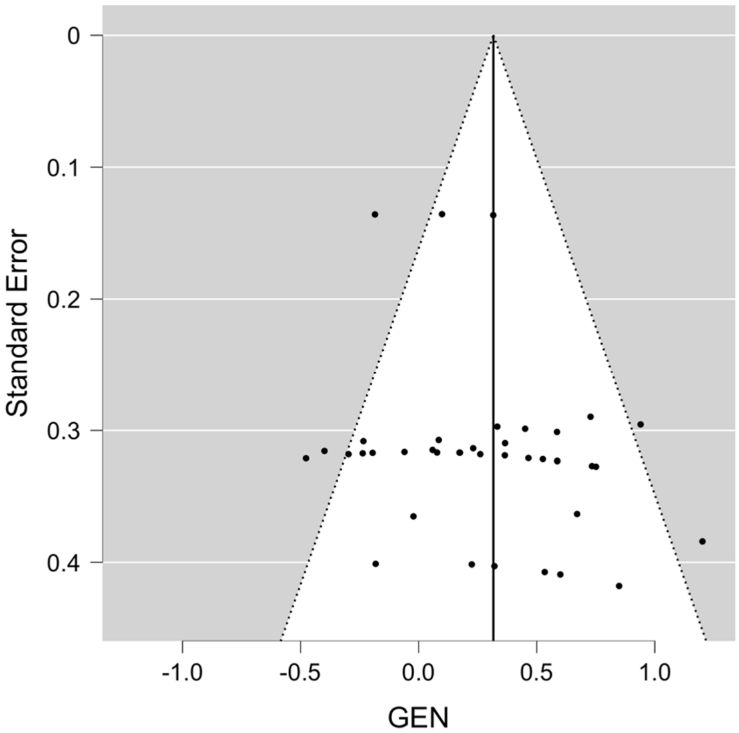

Rank Correlation test for funnel plot asymmetry shows no indication of publication bias (Kendall's tau=0.124, *p*=0.258)

Table S3. Descriptive Statistics of the comorbidity group (ADHD+ASD)

	ADHD+ASD (N=298)	
Variable	*Mean*	*SD*
Age	10.3	0.747
IQ	104	3.29
Females	32 (11%)	

Main Analyses: ADHD vs ADHD+ASD comparison

Comparing ADHD to the double diagnosis group resulted in a non-significant effect, $Z = 0.517$, $p = 0.605$, associated to a low Cohen's $d = 0.04$ ($SE = 0.08$, CI = [-0.12, 0.20]). This result meant that on the executive profile assessed through neuropsychological test, the ADHD group and the double diagnosis group did not differ. Test for the residual heterogeneity was still significant, $Q(36) = 94.507$, $p < .001$

Main Analyses: ASD vs ADHD+ASD comparison

On the contrary, comparing ASD to ADHD+ASD resulted in a significant effect, $Z = 3.648$, $p < .001$, $d = 0.22$ ($SE = 0.06$, CI = [0.101, 0.335]), meaning that, on neuropsychological test, the ASD group generally performed better than the double diagnosis group, with an effect size of small magnitude. The heterogeneity statistic was significant, $Q(36) = 53.456$, $p = 0.031$

Table S4. Results of the standardized differences between ASD, ADHD and ADHD+ASD.

	ADHD vs ADHD+ASD	ASD vs ADHD+ASD
d Global EFs	0.04	0.22***

Moderation Analyses

Table S5. Results of continuous moderators

Contrast	IQ Difference			Female Difference			Age Difference		
	Slope	Z	*p*	Slope	Z	*p*	Slope	Z	*p*
ADHD vs ADHD+ASD	0.015	0.729	0.466	0.086	1.825	0.068	-0.177	-1.844	0.06
ASD vs ADHD+ASD	-0.028	-1.599	0.11	3.048	0.012	0.991	-0.038	-0.337	0.736

Table S6. Results of categorical moderators

Contrast	Type of Publication *(Published vs Dissertation)*		
	Q	*d*	*p*
ADHD vs ADHD+ASD	0.316	0.02; 0.133	0.574
ASD vs ADHD+ASD	0.731	0.199; 0.157	0.393

Note. *Italic Text* indicates the levels of the categorical variable “type of publication”.
